# Recovery of Lithium and Cobalt from Spent Lithium-Ion Batteries Using a Deep Eutectic Solvent Based on Choline Chloride and Oxalic Acid (Oxaline)

**DOI:** 10.3390/molecules30244690

**Published:** 2025-12-07

**Authors:** Jessica M. Guamán-Gualancañay, Carlos F. Aragón-Tobar, Katherine Moreno, José-Luis Palacios, Diana Endara

**Affiliations:** 1 Department of Extractive Metallurgy, Escuela Politécnica Nacional, Ladrón de Guevara E11-253, P.O. Box 17-01-2759, Quito 170525, Ecuador; jessimguaman@gmail.com (J.M.G.-G.); carlos.aragont@epn.edu.ec (C.F.A.-T.); katherine.moreno@epn.edu.ec (K.M.); 2 Department of Mechanical Engineering, Escuela Politécnica Nacional, Ladrón de Guevara E11-253, P.O. Box 17-01-2759, Quito 170525, Ecuador; jose.palacios@epn.edu.ec

**Keywords:** deep eutectic solvent (DES), lithium-ion battery recycling, cobalt recovery, oxaline leaching

## Abstract

The growing consumption of lithium-ion batteries (LIBs) in electronic devices and electric vehicles has led to a significant increase in waste containing valuable metals such as lithium and cobalt. Recovering these metals is essential to reducing dependence on primary sources and minimizing environmental impact. In this study, the leaching of the cathode active material from discarded LIBs was evaluated using oxaline, a deep eutectic solvent (DES) composed of oxalic acid and choline chloride in a 1:1 molar ratio. The process began with the collection, discharge, washing, drying, and dismantling of the LIBs, followed by the separation of their components. Subsequently, the cathode active material was characterized, revealing a primary composition of cobalt (54.5%) and lithium (6.5%), with the presence of LiCoO_2_ confirmed by XRD analysis. Leaching experiments were conducted to evaluate the effects of temperature, time, and solid percentage, demonstrating that oxaline is effective for the selective leaching of lithium and cobalt. Under optimal conditions (90 °C, 1–2 wt.% cathode active material, 400 rpm), lithium underwent complete dissolution within the first hour, while cobalt achieved complete leaching by 4 h. Both metals were recovered as oxalates and separated based on differences in solubility. Oxaline proves to be an efficient and environmentally friendly alternative for the selective recovery of lithium and cobalt from LIB waste, supporting a circular economy in the management of critical metals.

## 1. Introduction

The use of lithium-ion batteries (LIBs) has expanded rapidly in recent years due to their high energy density, long lifespan, and electrochemical efficiency, features that have made them the primary power source for portable electronic devices and electric vehicles [1,2]. Global production is projected to reach 400,000 tons by 2025, resulting in a substantial volume of end-of-life batteries [3]. If not properly managed, these spent batteries become hazardous waste, releasing heavy metals and toxic compounds that pose serious environmental and public health risks [4,5]

The growing demand for LIBs has also raised concerns about the long-term sustainability of critical raw materials such as lithium and cobalt, key components of the cathode active material [6]. The extraction of these metals from primary sources involves considerable environmental and economic costs [7]. Cobalt, for example, is primarily obtained as a byproduct of nickel and copper mining, making its supply and pricing highly uncertain [8,9]. In parallel, the socio-environmental impacts associated with lithium extraction from South American brines present additional challenges to the sustainability of this resource [10].

Conventional recycling methods are typically based on pyrometallurgical and hydrometallurgical processes, often employing strong inorganic acids such as H_2_SO_4_ to achieve metal leaching efficiencies above 90% [11,12,13]. However, these methods are associated with significant drawbacks, including the generation of hazardous waste and high treatment costs [14]. As a more sustainable alternative, deep eutectic solvents (DESs) have gained attention in recent years for the recovery of metals. Oxaline, a biodegradable, low-toxicity mixture of oxalic acid and choline chloride, has shown promising results, with reported recovery efficiencies of 80–90% for Li and Co under mild conditions [15].

In this context, LIB recycling emerges as a viable strategy to recover valuable metals, extend material lifecycles, and mitigate pressure on natural resources [8,16]. Among the available technologies, leaching with deep eutectic solvents (DESs) has stood out as a promising option due to their selective dissolution capacity, low environmental impact, and alignment with green chemistry principles [17,18,19,20].

In recent years, numerous studies have explored the selective leaching of lithium and cobalt from spent lithium-ion batteries (LIBs) using choline chloride–based deep eutectic solvents (DESs), highlighting their potential as sustainable alternatives to conventional leaching agents. Tran et al. [18] first demonstrated the use of ethaline (a 1:2 molar ratio of choline chloride and ethylene glycol) for leaching LiCoO_2_ at 220 °C for 24 h, achieving over 90% Co and 85% Li extraction. The cobalt was subsequently recovered as Co_3_O_4_ via calcination. Wang et al. [21,22] later reported that similar extraction efficiencies could be obtained using reline (a 1:2 molar ratio of choline chloride and urea), which reduced the required temperature to 180 °C and halved the leaching time to 12 h. Schiavi et al. [23] achieved 90% Co and 10% Ni recovery with a choline chloride–ethylene glycol system, while Peeters et al. [24] obtained 81% Co recovery using choline chloride–citric acid at 80 °C for 6 h. Lu et al. [15] investigated a choline chloride–oxalic acid (oxaline) DES, achieving efficient Li and Co extraction (80–90%) under relatively mild conditions (90 °C, 2 h) using high-purity LiCoO_2_ (99%) as a model substrate, demonstrating the effectiveness of oxaline in promoting metal leaching at moderate temperatures. More recently, Li et al. [25] reported extremely rapid cobalt leaching, complete within only 10 s, also using a choline chloride–oxalic acid DES at 180 °C. Collectively, these studies highlight the critical influence of hydrogen bond donors (HBDs) on the efficiency and kinetics of metal recovery, underscoring the versatility, tunability, and potential energy savings of choline chloride–based DESs in LIB recycling.

This study aims to explore the technical feasibility of using oxaline, a deep eutectic solvent composed of choline chloride and oxalic acid, for the selective leaching and recovery of lithium and cobalt from spent lithium-ion batteries under varying operational conditions. The goal is to generate preliminary data on the leaching behavior of oxaline and assess its potential as a low-toxicity, sustainable alternative to conventional leaching agents. At the same time, the broader aim is to support circular-economy principles; complete economic and environmental feasibility assessments are considered outside the scope of this initial investigation.

## 2. Results

### 2.1. Chemical and Crystalline Phase Characterization of the Cathode Active Material from Lithium-Ion Batteries

The elemental chemical characterization of the calcined cathode active material, performed by X-ray Fluorescence (XRF), is presented in Table 1.

As shown in Table 1, cobalt (Co) is the predominant element, accounting for 54.5% of the content. Lithium (Li), determined by atomic absorption due to its low atomic weight, reached a content of 6.5% in the sample. Other elements such as manganese (Mn), nickel (Ni), and aluminum (Al) were found in lower proportions, with values of 9.8%, 1.5%, and 0.9%, respectively. These results confirm that the cathode active material is primarily composed of lithium cobalt oxide (LiCoO_2_), which was further corroborated by crystalline phase analysis.

**Figure 1:**
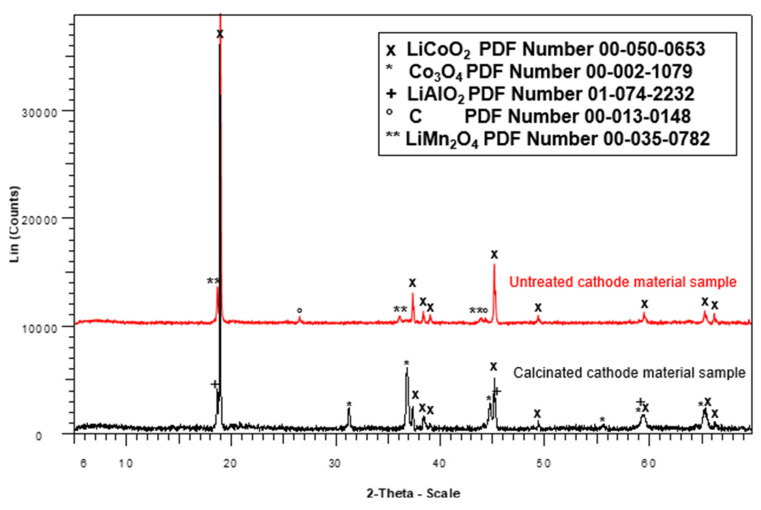
X-ray diffractogram of the cathode active material (untreated and calcinated).

Figure 1 presents the X-ray diffraction (XRD) patterns of the active material before and after calcination (700 °C, 5 h), a step performed to eliminate residual carbon from the electrode coating and to improve the crystallinity of the LiCoO_2_ phase.

Figure 1 reveals the predominance of lithium cobalt oxide as the main crystalline phase, along with secondary oxides such as cobalt oxide and lithium aluminum oxide, for the calcinated cathode active material. This is consistent with current literature [26,27,28,29], which confirms that lithium-ion batteries are primarily composed of lithium cobalt oxide as the cathode active material. The presence of secondary oxides, such as cobalt oxide and lithium aluminum oxide, in the analyzed sample may be attributed to the thermal treatment by calcination at 700 °C for 5 h, which can promote phase transformation and the formation of new oxides. The detection of aluminum in the form of LiAlO_2_ is likely not due to its presence in the active material itself but instead to migration from the aluminum current collector foil during mechanical separation. This migration could result in the incorporation of aluminum into the structure during the calcination process. Minor metals such as manganese and nickel were not detected in the crystalline phase identified by X-ray diffraction. This absence may be due to their dispersion within the major oxide matrix or their presence in concentrations below the detection limit of the Rietveld refinement method (typically ~1 wt.%). As such, although both metals are present in the chemical composition (Table 1), they do not form distinct crystalline phases detectable under the conditions used.

Phase identification of the calcined active cathode was performed using X-ray diffraction (XRD), revealing the main phases as LiCoO_2_ (PDF #00-050-0653) [30] and Co_3_O_4_ (PDF #00-002-1079) [30]. The present study focused on qualitative phase analysis to confirm successful leaching and recovery. However, the potential of Rietveld refinement techniques to quantify phase compositions and detect minor phases is recognized. Future work will include a more comprehensive crystallographic analysis employing such methods, as demonstrated in the recent literature [31].

### 2.2. Leaching Experiments with a Deep Eutectic Solvent Composed of Choline Chloride and Oxalic Acid

The leaching experiments with oxaline were conducted at two temperatures (60 and 90 °C), two cathode active material concentrations (1 wt.% and 2 wt.%), and over a time range of 0 to 6 h, while maintaining constant stirring at 400 rpm. In all cases, the cathode active material was leached directly after calcination.

#### 2.2.1. Influence of Solids Percentage on Lithium and Cobalt Recovery

**Figure 2:**
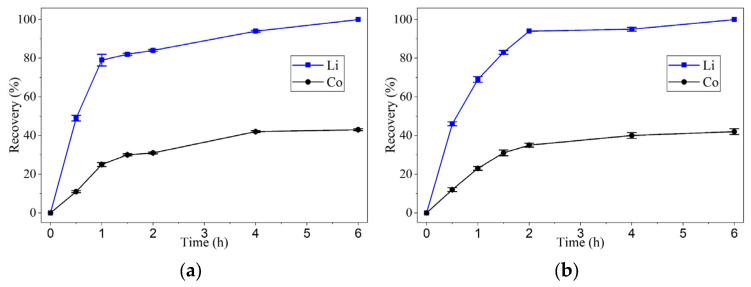
Leaching of calcined lithium-ion battery cathode active material using oxaline at 60 °C, 400 rpm, and 6 h, with two cathode active material concentrations: (**a**) 1 wt.% and (**b**) 2 wt.%.

Figure 2 presents the results of the leaching experiments carried out at 60 °C for 6 h under constant stirring at 400 rpm, using oxaline as the leaching agent. Two different cathode active material concentrations, 1 wt.% and 2 wt.%, were evaluated to assess their effect on the efficiency of lithium and cobalt extraction from the calcined cathode active material.

As shown in Figure 2a, at 60 °C and with a 1 wt.% cathode active material concentration, lithium reached a recovery value of 80% within 2 h and achieved complete recovery (100%) after 6 h. In contrast, cobalt exhibited lower dissolution rates, with recoveries of approximately 30% after two hours. By the end of the experiment, cobalt reached final recoveries of less than 40% (Figure 2a). When the cathode active material concentration was doubled to 2 wt.% under the same temperature conditions, the recovery of lithium exceeded 90% within 2 h. It stabilized near 100% after 6 h, while cobalt showed only a slight improvement, reaching just over 40% by the end of the test (Figure 2b). These results suggest that at the tested temperature of 60 °C, increasing the cathode active material concentration yields comparable recovery levels for the two studied metals.

#### 2.2.2. Influence of Temperature on Lithium and Cobalt Recovery

**Figure 3:**
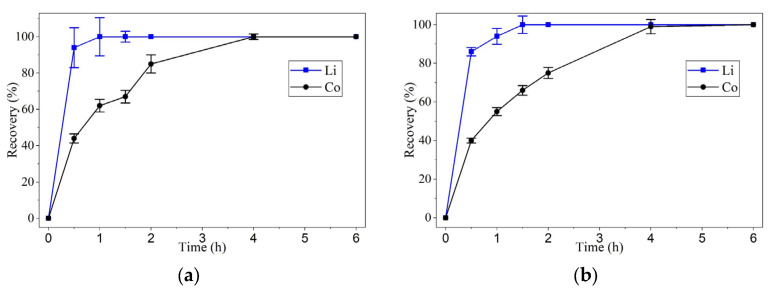
Leaching of calcined lithium-ion battery cathode active material using oxaline at 90 °C, 400 rpm, and 6 h, with two cathode active material concentrations: (**a**) 1 wt.% and (**b**) 2 wt.%.

Figure 3 presents the results of the leaching experiments carried out at 90 °C for 6 h under constant stirring at 400 rpm, using oxaline as the leaching agent. Two different cathode active material concentrations, 1 wt.% and 2 wt.%, were evaluated to assess their effect on the efficiency of lithium and cobalt extraction from the calcined cathode active material.

As shown in Figure 3a, at 90 °C with 1 wt.% cathode active material concentration, lithium underwent complete dissolution within the first hour. Cobalt exhibited a significant increase in recovery, reaching 80% at 2 h and achieving complete leaching by 4 h. Under the same temperature (90 °C) but with a 2 wt.% cathode active material concentration, both metals reached complete recovery in less than 6 h. Lithium was fully recovered in just 1.5 h, while cobalt achieved complete recovery at 4 h, confirming that the combination of higher temperature and cathode active material concentration enhances metal extraction efficiency (Figure 3b).

Preliminary experiments were also performed at 30 °C and with five wt.% solids to evaluate the effect of viscosity on the leaching process. At 30 °C, the system exhibited high viscosity, which hindered proper mixing and mass transfer, resulting in limited metal dissolution. Similarly, at higher solid loadings (5 wt.%), the viscosity of the DES increased significantly, rendering the mixture unmanageable even at temperatures as high as 60 °C and 90 °C. Therefore, the selected operational range (60–90 °C and 1–2 wt.% solids) represents the most practical and reproducible window for efficient leaching using oxaline.

During the leaching experiments conducted under optimized conditions (90 °C, 1–2 wt.%, 400 rpm), no solid residue was observed after filtration, indicating complete dissolution of the cathode material in the deep eutectic solvent. This was consistent with the near-complete recovery of lithium and cobalt. As a result, no leaching residues were available for further structural or morphological characterization.

### 2.3. Recovery of Lithium and Cobalt as Oxalates from the Leachate

**Figure 4:**
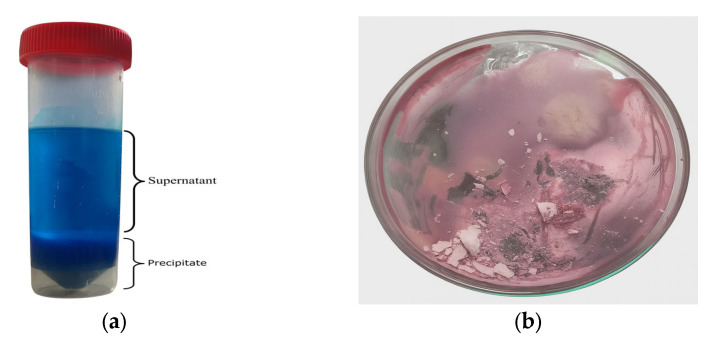
(**a**) Separation of the supernatant and precipitate after ethanol washing of the oxaline leachate. (**b**) Dried precipitate obtained after removal of residual ethanol and oxaline.

After the leaching process was completed, the resulting DES was first washed with ethanol to separate the supernatant from the precipitate, as shown in Figure 4a. The collected precipitate was then dried to remove residual liquid of ethanol and oxaline. The dry solid is shown in Figure 4b.

Figure 4a illustrates the separation of the supernatant and precipitate following ethanol washing of the leachate. The supernatant is expected to contain ethanol and oxaline, which can be evaporated to remove ethanol and isolate the remaining oxaline. Although the efficiency and characterization of the recovered oxaline in subsequent leaching cycles were not evaluated in this study, it presents a promising avenue for future research aimed at improving the sustainability and economic viability of cobalt and lithium recovery processes using oxaline-based deep eutectic solvents.

**Figure 5:**
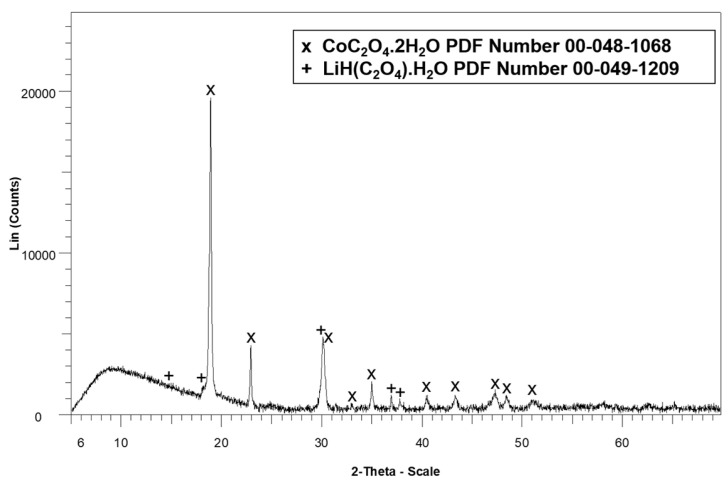
X-ray diffractogram of the precipitate obtained after ethanol washing.

To further investigate the nature of the recovered solids, the precipitate shown in Figure 4b was analyzed using X-ray diffraction (XRD), with the results presented in Figure 5. In the precipitate obtained after ethanol washing, both lithium and cobalt were present in the form of their respective oxalates (CoC_2_O_4_·2H_2_O and LiH(C_2_O_4_)·H_2_O).

#### 2.3.1. Recovery of Lithium Precipitates

**Figure 6:**
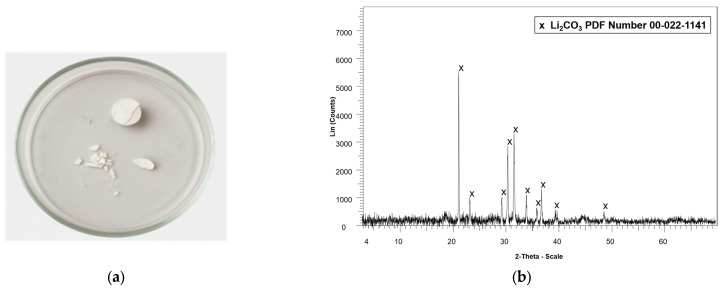
Lithium compound recovered (**a**) after evaporation of the aqueous solution and (**b**) X-ray diffractogram of the lithium precipitate after calcination (500 °C and 2 h).

The solid residue obtained after ethanol washing was then subjected to an additional washing step with water to separate lithium oxalate (which is water-soluble) from cobalt oxalate (which remains as a precipitate), based on their differences in solubility. The aqueous lithium solution was subsequently evaporated and calcinated to yield a solid lithium compound, as presented in Figure 6a.

Lithium was successfully recovered as a white powder, as shown in Figure 6a. In this study, 0.08 g of a lithium-containing compound was recovered from 0.25 g of the leached sample (calcined active material). This solid obtained after evaporation was subsequently calcinated at 500 °C for two hours. The XRD pattern (Figure 6b) shows the characteristic peaks of lithium carbonate, confirming recovery of this phase. In addition, the resulting sample was acid-digested and analyzed by atomic absorption spectroscopy (AAS) to quantify the lithium content. The analysis indicated that the precipitate contains approximately 15% lithium, confirming the presence of lithium in the white powder.

#### 2.3.2. Recovery of Cobalt Precipitates

**Figure 7:**
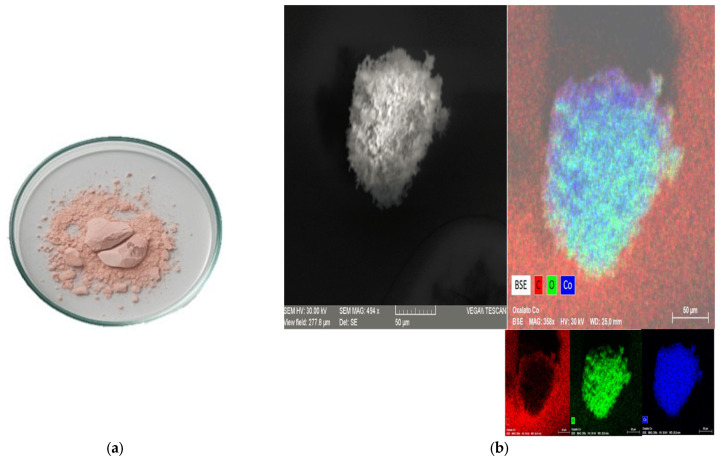
Cobalt compound recovered. (**a**) Pink-colored cobalt-containing precipitate obtained after the selective dissolution of lithium. (**b**) Characterization by SEM-EDS of the cobalt precipitate.

The solid obtained after the ethanol wash was then rinsed with water to dissolve the lithium oxalate, leaving behind a pink-colored, cobalt-containing precipitate, as shown in Figure 7a. This precipitate was characterized by Scanning Electron Microscopy and Energy-Dispersive X-ray Spectroscopy (SEM-EDS), as presented in Figure 7b.

**Figure 8:**
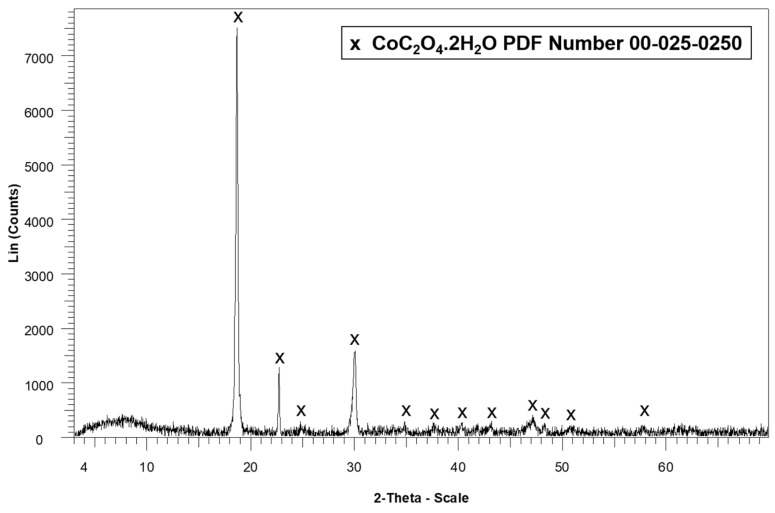
X-ray diffractogram of the cobalt precipitate obtained after selective dissolution of lithium.

The powder was analyzed by X-ray diffraction (XRD) to confirm the presence of hydrated cobalt oxalate (CoC_2_O_4_·2H_2_O), as shown in the diffractogram in Figure 8. The XRD pattern shows the characteristic peaks of hydrated cobalt oxalate, confirming recovery of this phase. From 0.25 g of the leached sample (cathode active material), 0.12 g of hydrated cobalt oxalate was recovered as a pink-colored powder. These results indicate that the precipitation method was effective not only in recovering cobalt in the form of oxalate but also in successfully separating it from lithium, which remained in solution and was recovered separately, as presented previously.

## 3. Discussion

### 3.1. Lithium and Cobalt Dissolution Behavior with Oxaline

Lithium exhibited rapid leaching kinetics, reaching recovery values above 80% within the first 2 h, regardless of the two temperatures tested. At 90 °C, its dissolution was virtually instantaneous within the first 60 min.

Cobalt, in contrast, showed significantly slower kinetics, particularly at 60 °C, where recovery plateaued around 40–50%. However, at 90 °C, the dissolution rate increased substantially, achieving 80% recovery in 2 h and complete leaching within 4 h. This behavior suggests that cobalt dissolution is strongly influenced by thermal energy, possibly due to the need to break the crystalline structure of LiCoO_2_ and form soluble complexes with oxalic acid.

These results indicate that temperature is the most critical factor affecting leaching kinetics, especially for cobalt. At 90 °C, oxaline demonstrated a significant capacity for the selective dissolution of the target metals, with lithium being the most readily leached, followed by cobalt. One of the most important findings of this study was oxaline’s high selectivity toward lithium. At 90 °C, lithium was dissolved entirely within 1 h, while cobalt required 4 h to reach the same recovery level. This difference in leaching kinetics suggests the potential for a stage-wise, selective separation process, allowing for optimization of both time and conditions for each metal.

Considering the optimal experimental conditions identified in this study using oxaline (90 °C and 6 h), a comparative analysis was conducted with previously reported processes. For instance, Tran et al. [18] employed ethaline and achieved extraction efficiencies above 90% for Co and 85% for Li; however, this required relatively harsh conditions (220 °C and 24 h), significantly higher in both temperature and duration compared to our study. Wang et al. [21,22] improved upon this by replacing ethaline with reline, thereby reducing the required temperature to 180 °C and halving the leaching time, while maintaining comparable recovery rates, albeit under more severe thermal conditions. Similarly, Schiavi et al. [23] used ethaline to recover approximately 90% cobalt at 180 °C over 24 h, again under considerably harsher conditions than those applied in our work. In contrast, Peeters et al. [24] employed a deep eutectic solvent (DES) composed of choline chloride and citric acid, achieving 81% cobalt recovery after 6 h at 80 °C, conditions quite similar to those of the present study. These comparisons underscore the crucial role of the hydrogen bond donor (HBD) in determining the efficiency and environmental sustainability of DES-based leaching systems. The results suggest that oxalic acid, as an HBD, offers distinct advantages due to its dual function as both a complexing and reducing agent, enhancing metal solubilization under relatively mild conditions. Consequently, the use of oxaline demonstrates that careful selection of the HBD can markedly improve the performance of DES, reducing energy requirements while maintaining high recovery yields.

Regarding the use of oxaline (a 1:1 mixture of oxalic acid and choline chloride), Lu et al. [15] investigated this DES. They achieved efficient extraction of Li and Co (80–90%) under relatively mild conditions (90 °C, 2 h) using pure LiCoO_2_ (99%) as a model substrate. Their work highlighted the role of oxalic acid as a hydrogen bond donor (HBD) in enabling high metal recovery at lower temperatures, suggesting a more energy-efficient approach to LIB recycling. In comparison, our study achieved similar leaching efficiencies using real spent LIB cathode material, which typically contains additional metallic oxides and structural degradation, highlighting the practical applicability and robustness of oxaline under more realistic recycling conditions.

Li et al. [25] reported exceptionally rapid cobalt leaching using a choline chloride–oxalic acid DES at 180 °C, achieving nearly complete recovery within only 10 s. While these results demonstrate the high reactivity of oxalic acid as a hydrogen bond donor (HBD) at elevated temperatures, the process still requires considerably harsher thermal conditions compared to the present study (90 °C, 6 h). In our work, comparable cobalt recovery was achieved under much milder conditions, emphasizing the potential of oxaline to maintain high leaching efficiency without the need for extreme temperatures or pressures. This comparison highlights the versatility of oxalic acid in DES formulations. It underscores that, although reaction kinetics can be accelerated at higher temperatures, efficient leaching can also be achieved through optimized solvent composition at moderate conditions. Consequently, the results from both studies collectively demonstrate the strong coordination and reducing capabilities of oxalic acid as an HBD; however, our findings reveal that these advantages can be effectively harnessed in a more energy-efficient and environmentally sustainable manner.

### 3.2. Manganese and Nickel Dissolution Behavior with Oxaline

Nickel and manganese were also detected in the active material of the cathode, as shown in Table 1, and their concentrations were also monitored during the leaching experiments. However, a detailed analysis of the dissolution behavior of these metals falls outside the scope of the present study, which focuses specifically on the leaching efficiency of cobalt and lithium using oxaline. Including a broader range of metals, such as nickel and manganese, would provide a more comprehensive understanding of the leaching of oxaline capabilities and is recommended for future research. It is essential to consider that the concentrations of nickel and manganese in cathode materials vary considerably depending on the battery chemistry, manufacturer, and generation. In our study, ~9.8% Mn and ~1.5% Ni were measured in our batch of batteries, values that reflect only the particular sample set. By contrast, many commercially used lithium—nickel—manganese—cobalt (NMC) cathodes contain substantially higher nickel and manganese contents. For example, NMC—111 (LiNi_0.33_Mn_0.33_Co_0.33_O_2_) has approximately 33% Ni and 33% Mn by molar fraction [32]. In more nickel—rich formulations, such as NMC—811, nickel constitutes ~80% of the transition metal content, with far lower proportions of manganese and cobalt [33]. These significant differences suggest that our measured values are on the lower end for Mn and Ni compared to many commercially essential cathode materials.

Because of this variability, focusing only on cobalt and lithium in this work is reasonable given our sample’s composition. However, for battery types with higher Ni or Mn content, the leaching behavior of these metals could be considerably more relevant. Including Ni and Mn in future studies would therefore help establish whether the selectivity and extraction kinetics of oxaline are affected by their presence, particularly for modern nickel—rich battery chemistries.

### 3.3. Future Perspectives on DES-Based Recovery of Lithium and Cobalt

The present study demonstrates that oxaline, a deep eutectic solvent (DES) composed of choline chloride and oxalic acid, provides an efficient and selective medium for recovering lithium and cobalt from spent lithium-ion batteries (LIBs). Building on these promising findings, further research is required to advance oxaline-based leaching from laboratory validation to practical, sustainable implementation. Key areas for future investigation include material regeneration, mechanistic understanding, and process scalability.

While this study successfully demonstrates the selective leaching and recovery of lithium and cobalt using oxaline, further work is required to evaluate the potential reuse of the recovered materials. Specifically, the re-synthesis or re-calcination of recovered precursors to regenerate LiCoO_2_ with its original layered structure is a critical step toward establishing a closed-loop recycling process. Future studies will focus on the structural regeneration and electrochemical performance of the reconstituted cathode materials to assess their suitability for reuse in lithium-ion batteries.

Further insight into the leaching mechanism and molecular interactions within oxaline is also needed. Advanced characterization techniques, such as X-ray Photoelectron Spectroscopy (XPS), Nuclear Magnetic Resonance (NMR) spectroscopy, speciation analysis, and kinetic modeling, should be employed to elucidate oxidation states, coordination environments, and the role of hydrogen bonding in metal dissolution. These studies will contribute to a deeper understanding of how the composition and structure of DESs govern leaching efficiency and selectivity, providing a foundation for the rational design of next-generation DESs for metal recovery.

In addition, evaluating the economic and environmental performance of oxaline-based recycling is essential to determine its industrial feasibility. Future work should include a comprehensive cost–benefit analysis, life cycle assessment (LCA), and energy input–output modeling to quantify the process’s sustainability advantages relative to conventional approaches, such as inorganic acid leaching, pyrometallurgical processing, and bioleaching. Such assessments will help position DES-based systems as viable alternatives for large-scale recycling of critical metals.

Finally, process optimization and scale-up studies are necessary to bridge the gap between laboratory research and industrial application. The development of continuous or semi-continuous leaching systems, supported by process simulation and reactor engineering, will enable the evaluation of operational stability, solvent recyclability, and energy efficiency. Currently, limited studies examine the techno-economic and environmental metrics of DES-based recycling routes, underscoring the novelty and importance of advancing this research direction.

## 4. Materials and Methods

### 4.1. Chemical and Crystalline Phase Characterization of LIB Components

A total of 1594.8 g of lithium-ion batteries (LIBs) were collected from discarded cell phones as part of a recycling campaign conducted at Escuela Politécnica Nacional, Quito, Ecuador). The LIBs were collected and weighed; the voltage of each was measured, and then they were discharged under the following conditions: a 5 vol.% NaCl saline solution for 24 h [34,35]. After this period, the voltage was measured again to ensure the battery was fully discharged. The batteries were then washed and dried at 110 °C to remove any moisture [36]. Subsequently, the batteries were dismantled, and their components (metal casing, cathode, anode, and plastic separator) were separated. From the components obtained during dismantling, only the cathode and its active material were selected for further processing and analysis. The valorization of the remaining battery components lies beyond the scope of this article but represents an ongoing line of investigation by the research team presenting this study.

The cathode, composed of an aluminum foil coated with an active material identified as LiCoO_2_, was heated in a muffle furnace at 300 °C for 30 min to facilitate the separation of the aluminum foil from the active material [36]. The recovered active material, containing LiCoO_2_, was then calcined at 700 °C for 5 h to remove residual carbon and other impurities [20,36]. Crystalline phase characterization was performed using X-ray diffraction (XRD) with a Bruker AXS D8 ADVANCE diffractometer (Bruker, Karlsruhe, Germany), which enabled the identification of crystalline phases containing lithium and cobalt in the LIB cathode material.

The chemical composition of the elements present in the LIBs was determined by X-ray fluorescence (XRF) using a Bruker S8 Tiger instrument (Bruker, Karlsruhe, Germany). Since XRF can only detect elements from the third period onward, lithium (Li) was quantified separately using atomic absorption spectroscopy (AAS) after acid digestion of the powdered sample.

For chemical characterization, the powder obtained from the calcined cathode was digested using an acid digestion method. A 0.05 g sample was placed in Teflon beakers containing 10 mL of deionized water and heated for 5 min. Then, 10 mL of analytical-grade hydrochloric acid (HCl 36.5–38%, Fisher, Geel, Belgium) was added, and the mixture was boiled for 30 min. Afterward, 10 mL of nitric acid (HNO_3_ 70%, Fisher) and 2 mL of perchloric acid (HClO_4_ 60–62%, JT Baker, Phillisburg, NJ, USA) were added. The solution was evaporated to dryness until no white fumes were observed. Once the white fumes had dissipated, 10 mL of the mixture in a 3:1 ratio (HNO_3_:HCl) was heated until complete dissolution of the sample was achieved [37]. After cooling, the digested sample was transferred to a 100 mL volumetric flask and diluted to volume for lithium analysis by atomic absorption spectroscopy using a PerkinElmer 300 instrument.

### 4.2. Preparation of Oxaline

For the preparation of the deep eutectic solvent named oxaline, the starting reagents were first dried to eliminate residual moisture. Oxalic acid (C_2_H_2_O_4_, ≥99.0% purity, Sigma-Aldrich, Stinheim, Germany), serving as the hydrogen bond donor (HBD), and choline chloride (ChCl, 98% purity, Sigma-Aldrich, Stinheim, Germany), acting as the hydrogen bond acceptor (HBA), were both dried in an oven at 70 °C for 1 h. Following this, oxaline was prepared by mixing choline chloride and oxalic acid in a 1:1 molar ratio.

### 4.3. Leaching Experiments with Oxaline and the Calcined Active Material from LIBs

Four leaching experiments were carried out using oxaline, a deep eutectic solvent (DES) composed of choline chloride and oxalic acid in a 1:1 molar ratio. The leaching target was the calcined active material recovered from the cathodes of spent lithium-ion batteries (LIBs). Each experiment was conducted in a sealed glass flask by mixing the cathode active material with 25 g of oxaline. Two sets of experiments were performed at 60 °C and two at 90 °C, each at cathode active material concentrations of 1 wt.% and 2 wt.%. For the 1 wt.% cathode active material concentration, 0.25 g of calcined material was used, and for the 2 wt.% concentration, 0.5 g was added. The temperature during the leaching process was regulated using a glycerin bath, and the mixtures were continuously stirred at 400 rpm throughout the experiments.

To monitor the leaching experiments, 200 μL aliquots were collected at 0, 0.5, 1, 1.5, 2, 4, and 6 h. Each aliquot was filtered and diluted with a 2 vol.% nitric acid solution before analysis by atomic absorption spectroscopy (PerkinElmer Analyst 300, Shelton, CT, USA) to quantify the concentrations of lithium (Li) and cobalt (Co). No solid residue was observed after the leaching process, indicating that the cathode material had been completely dissolved.

Each experimental point presented in Figure 2 and Figure 3 corresponds to the average of three independent leaching tests carried out under identical conditions to ensure reproducibility. The concentrations of lithium and cobalt in the leachate were determined by atomic absorption spectroscopy (AAS) after solid–liquid separation. The standard deviation (SD) of the measurements was calculated and found to be within ±11% for lithium and ±4% for cobalt recovery, indicating acceptable experimental reproducibility. Error bars in Figure 2 and Figure 3 represent the calculated SD values for each data point.

### 4.4. Recovery of Lithium and Cobalt Dissolved in Oxaline via Precipitation

Once the DES enriched in lithium (Li) and cobalt (Co) was obtained, 10 mL of ethanol was added, and the mixture was centrifuged using a SIGMA 2-6 centrifuge at 4000 rpm for 1 h. The resulting precipitate and supernatant were separated. The supernatant was subjected to a second centrifugation under the same conditions to maximize the recovery of dissolved metals. After this second centrifugation, the resulting supernatant was left to rest for 24 h to allow further precipitation of any remaining Li and Co species. The final supernatant was then evaporated to recover the DES.

All precipitates collected from the centrifugation and decantation steps were dried at 40 °C and subsequently washed four times with 10 mL of deionized water to separate the more soluble lithium compounds from the less soluble cobalt compounds. This process resulted in two distinct phases: a solid phase consisting predominantly of cobalt compounds, and a supernatant containing lithium in solution. The lithium-containing supernatant was evaporated at 110 °C in a laboratory oven for 2 h to recover lithium as a solid precipitate, which was subsequently calcined at 500 °C for 2 h. The cobalt-rich precipitate was dried at 110 °C for 2 h in a laboratory oven to remove residual moisture.

Both solid products were analyzed by X-ray diffraction (XRD) to identify their crystalline phases. Additionally, the residual concentrations of Li and Co in the wash solutions were quantified using atomic absorption spectroscopy (AAS) with a PerkinElmer AAnalyst 300 instrument (PerkinElmer, Shelton, CT, USA). Furthermore, 0.05 g of each dried precipitate was digested and analyzed by AAS to verify the content of Li and Co in the recovered solids. Additionally, the presence of metals such as cobalt in the precipitates was analyzed by scanning electron microscopy coupled with energy-dispersive X-ray spectroscopy (SEM–EDS) using a Tescan Vega LMU instrument (TESCAN, Brno, Czech Republic).

## 5. Conclusions

The active material of the cathode was successfully recovered from spent lithium-ion batteries (LIBs) through mechanical separation from other LIB components, including copper, graphite, and aluminum. Chemical and crystalline phase characterization of this material revealed that lithium (Li) and cobalt (Co) were the predominant elements, with concentrations of 6.5% and 54.5%, respectively, followed by manganese (Mn) at 9.8% and nickel (Ni) at 1.5%. Other minor elements were present in concentrations of less than 1%. X-ray diffraction (XRD) analysis of the calcined active material confirmed the presence of cobalt oxide (CoO) and lithium aluminum oxide (LiAlO_2_).

Leaching experiments using oxaline, a deep eutectic solvent (DES) composed of choline chloride and oxalic acid in a 1:1 ratio, were conducted at two temperatures (60 °C and 90 °C) and two cathode active material concentrations (1 wt.% and 2 wt.%). At 60 °C and 1 wt.%, lithium exhibited recovery efficiencies greater than 80% within 2 h, reaching nearly 100% after 6 h. In contrast, cobalt showed more moderate recoveries, remaining below 50% after 6 h. At 90 °C and 1 wt.% cathode active material concentrations, lithium reached complete recovery within 2 h, while cobalt achieved approximately 80% recovery under the same conditions, highlighting the strong influence of temperature on leaching efficiency. Notably, at 90 °C and 1 wt.% cathode active material concentration, both lithium and cobalt reached complete recovery after 4 h of leaching with oxaline. Increasing the solid percentage from 1 to 2 wt.% did not significantly affect the recovery efficiencies for either metal.

This study successfully demonstrated the selective recovery of lithium and cobalt from spent lithium-ion battery cathodes using oxaline as a deep eutectic solvent. The leaching process achieved near-complete dissolution of target metals under mild conditions. Furthermore, the subsequent selective precipitation approach enabled efficient separation of lithium and cobalt compounds, contributing to a promising green and sustainable recycling route. These findings support the potential of oxaline-based processes to advance circular economy strategies in critical metal recovery.

## Figures and Tables

**Table 1:** Chemical characterization of the cathode active material.

Element	Content (%)
Co	54.5
Mn	9.8
Li *	6.5
Ni	1.5
Al	<1.0
P	<1.0
Other minor elements	<1.0

* Determined by atomic absorption.

## Data Availability

The original contributions presented in this study are included in the article. Further inquiries can be directed to the corresponding author.

## References

[1] López Hernández V., Hilbert I., Gascón Castillero L., Manhart A., García D., Nkongdem B., Dumitrescu R., Sucre C., Ferreira Herrera C. (2024). Reciclaje y reúso de baterías de litio en América Latina y el Caribe: Revisión analítica de prácticas globales y regionales. Recycling and Reuse of Lithium Batteries in Latin America and the Caribbean: Analytical Review of Global and Regional Practices.

[2] Quintero V., Che O., Ching E., Auciello O., De Obaldía E. (2021). Baterías de iones de litio: Características y aplicaciones Lithium ion batteries: Features and applications Universidad de Texas en Dallas. Rev. I+D Tecnológico.

[3] Gurudev S. El litio de las Baterías es la Sustancia que Mueve el Mundo: Cuánto sabes de este Metal. National Geographic 2024. https://www.nationalgeographic.es/medio-ambiente/2024/01/litio-que-es-importancia-baterias-elemento-mueve-mundo-metal.

[4] Mendoza A. Componentes y Periféricos: La Importancia de la Tecnología y las Computadoras en Nuestra Vida Diaria. https://trecebytes.com/tecnologia-y-computadoras/.

[5] UNEP—United Nations Environment Program (2024). Perspectiva Mundial de la Gestión de Residuos. https://www.unep.org/es/resources/perspectiva-mundial-de-la-gestion-de-residuos-2024.

[6] Xu C., Dai Q., Gaines L., Hu M., Tukker A., Steubing B. (2020). Future material demand for automotive lithium-based batteries. Commun. Mater..

[7] Lèbre É., Stringer M., Svobodova K., Owen J.R., Kemp D., Côte C., Arratia-Solar A., Valenta R.K. (2020). The social and environmental complexities of extracting energy transition metals. Nat. Commun..

[8] Weidenkaff A. (2019). Little precious lithium?. MRS Bull..

[9] Swain B. (2017). Recovery and recycling of lithium: A review. Sep. Purif. Technol..

[10] Romero-Carrión V.L., Ccasani-Allende J., Rivadeneyra-Rivas C.A., Altamirano-Romero J.C.G. (2023). Prospects of the use of lithium-ion battery vehicles and sustainable development in South America. Rev. Kawsaypacha Soc. Medioambiente.

[11] Davis K., Demopoulos G.P. (2023). Hydrometallurgical recycling technologies for NMC Li-ion battery cathodes: Current industrial practice and new R&D trends. RSC Sustain..

[12] Lombardo G., Ebin B., St Foreman M.R.J., Steenari B.M., Petranikova M. (2019). Chemical Transformations in Li-Ion Battery Electrode Materials by Carbothermic Reduction. ACS Sustain. Chem. Eng..

[13] Armas S., Endara D. (2017). Acid Leaching and Precipitation of Metals from Spent Li-ion Batteries. Proceedings of the EDP Congress 2017.

[14] Toro L., Moscardini E., Baldassari L., Forte F., Falcone I., Coletta J., Toro L. (2023). A Systematic Review of Battery Recycling Technologies: Advances, Challenges, and Future Prospects. Energies.

[15] Lu Q., Chen L., Li X., Chao Y., Sun J., Ji H., Zhu W. (2021). Sustainable and Convenient Recovery of Valuable Metals from Spent Li-Ion Batteries by a One-Pot Extraction Process. ACS Sustain. Chem. Eng..

[16] Greim P., Solomon A.A., Breyer C. (2020). Assessment of lithium criticality in the global energy transition and addressing policy gaps in transportation. Nat. Commun..

[17] Abbott A.P., Capper G., Davies D.L., McKenzie K.J., Obi S.U. (2006). Solubility of metal oxides in deep eutectic solvents based on choline chloride. J. Chem. Eng..

[18] Tran M.K., Rodrigues M.T.F., Kato K., Babu G., Ajayan P.M. (2019). Deep eutectic solvents for cathode recycling of Li-ion batteries. Nat. Energy.

[19] Marcus Y. (2019). Deep Eutectic Solvents.

[20] Valenzuela L., Quintana M., Rojas S., Segovia R. (2022). Reciclaje de Baterías de Litio: Una Realidad para la Electromovilidad de Chile. Report.

[21] Wang S., Zhang Z., Lu Z., Xu Z. (2020). A novel method for screening deep eutectic solvent to recycle the cathode of Li-ion batteries. Green Chem..

[22] Wang K., Hu T., Shi P., Min Y., Wu J., Xu Q. (2022). Efficient Recovery of Value Metals from Spent Lithium-Ion Batteries by Combining Deep Eutectic Solvents and Co-extraction. ACS Sustain. Chem. Eng..

[23] Schiavi P.G., Altimari P., Branchi M., Zanoni R., Simonetti G., Navarra M.A., Pagnanelli F. (2021). Selective recovery of cobalt from mixed lithium-ion battery wastes using deep eutectic solvent. Chem. Eng. J..

[24] Peeters N., Binnemans K., Riaño S. (2020). Solvometallurgical recovery of cobalt from lithium-ion battery cathode materials using deep-eutectic solvents. Green Chem..

[25] Li T., Xiong Y., Yan X., Hu T., Jing S., Wang Z., Ge X. (2022). Closed-loop cobalt recycling from spent lithium-ion batteries based on a deep eutectic solvent (DES) with easy solvent recovery. J. Energy Chem..

[26] Koech A., Mwandila G., Mulolani F., Mwaanga P. (2024). Lithium-ion battery fundamentals and exploration of cathode materials: A review. S. Afr. J. Chem. Eng..

[27] Huang Y. (2022). The discovery of cathode materials for lithium-ion batteries from the view of interdisciplinarity. Interdiscip. Mater..

[28] Fu W., Wang Y., Kong K., Kim D., Wang F., Yushin G. (2023). Materials and Processing of Lithium-Ion Battery Cathodes. Nanoenergy Adv..

[29] Wang J., Zhang Q., Sheng J., Liang Z., Ma J., Chen Y., Zhou G., Cheng H.M. (2022). Direct and green repairing of degraded LiCoO_2_ for reuse in lithium-ion batteries. Nat. Sci. Rev..

[30] (2015). Powder Diffraction File™ for Materials Characterization: LiCoO2—PDF #00-050-0653; LiMn2O4—PDF #00-035-0782; C—PDF #00-013-0148; Co3O4—PDF #00-002-1079; LiAlO2—PDF #01-074-2232; CoC2O4·2H2O—PDF #00-048-1068; LiH(C2O4)·2H2O—PDF #00-049-1209; CoC2O4·2H2O—PDF #00-025-0250; Li2CO3—PDF #00-022-1141.

[31] Guan D., Shi C., Xu H., Gu Y., Zhong J., Sha Y., Hu Z., Ni M., Shao Z. (2023). Simultaneously mastering operando strain and reconstruction effects via phase-segregation strategy for enhanced oxygen-evolving electrocatalysis. J. Energy Chem..

[32] Wang K., Weniger F., Berglund J., Nyholm L., Forsén O. (2019). Investigation of the leaching mechanism of NMC 811 (LiNi_0.8_Mn_0.1_Co_0.1_O_2_) by hydrochloric acid for recycling lithiumion battery cathodes. RSC Adv..

[33] Kaur A., Alarco J., O’ Mullane A.P. (2024). Investigating the Potential Use of NiMnCo (NMC) Battery Materials as Electrocatalysts for Electrochemical Water Splitting. ChemPhysChem.

[34] Li J., Wang G., Xu Z. (2016). Generation and detection of metal ions and volatile organic compounds (VOCs) emissions from the pretreatment processes for recycling spent lithium-ion batteries. Waste Manag..

[35] Wang F., Zhang T., He Y., Zhao Y., Wang S., Zhang G., Zhang Y., Feng Y. (2018). Recovery of valuable materials from spent lithium-ion batteries by mechanical separation and thermal treatment. J. Clean. Prod..

[36] Sarkar M., Hossain R., Sahajwalla V. (2024). Sustainable recovery and resynthesis of electroactive materials from spent Li-ion batteries to ensure material sustainability. Resour. Conserv. Recycl..

[37] Mashale K.N., Sehata J., Ntsasa N.G., Chimuka L., Tshilongo J. (2025). Chemical analysis of low grade gold from mine tailings after size fractionation and acid digestion using reverse aqua regia. Sci. Rep..

